# Cycling and Running are More Predictive of Overall Race Finish Time than Swimming in IRONMAN^®^ Age Group Triathletes

**DOI:** 10.1186/s40798-025-00835-8

**Published:** 2025-03-28

**Authors:** Beat Knechtle, David Valero, Elias Villiger, Mabliny Thuany, Ivan Cuk, Pedro Forte, Marilia Santos Andrade, Pantelis T. Nikolaidis, Thomas Rosemann, Katja Weiss

**Affiliations:** 1 Medbase St. Gallen Am Vadianplatz, Vadianstrasse 26, St. Gallen, 9001 Switzerland; 2 Institute of Primary Care, University of Zurich, Zurich, Switzerland; 3 Ultra Sports Science Foundation, Pierre-Benite, France; 4 Department of Physical Education, State University of Para, Pará, Brazil; 5 Faculty of Sport and Physical Education, University of Belgrade, Belgrade, Serbia; 6 CI-ISCE, Higher Institute of Educational Sciences of the Douro, Penafiel, Portugal; 7 Instituto Politécnico de Bragança, Bragança, Portugal; 8 Research Center in Sports, Health and Human Development, Covilhã, Portugal; 9 Department of Physiology, Federal University of Sao Paulo, Sao Paulo, Brazil; 10 School of Health and Caring Sciences, University of West Attica, Athens, Greece

**Keywords:** Master athlete, Prediction, Multi-sport, Aging, Triathlon

## Abstract

**Background:**

Several studies have evaluated the most predictive discipline (swimming, cycling, and running) of performance in elite IRONMAN^®^ triathletes. However, no study has ever determined the most decisive discipline for IRONMAN^®^ age group triathletes. The present study analyzed the importance of the three disciplines on the overall race times in IRONMAN^®^ age group triathletes, in order to try and determine the most predictive discipline in IRONMAN^®^ for age group triathletes, and whether the importance of the split disciplines changes with increasing age.

**Methods:**

This cross-sectional study used 687,696 IRONMAN^®^ age group triathletes race records (553,608 from males and 134,088 from females). Age group athletes were divided in 5-year age groups (i.e., 18–24, 25–29, 30–34,…,70–74, and last 75 + years). The relationships between split disciplines (i.e., swimming, cycling, and running) and overall race times were evaluated using Spearman and Pearson correlations. A multi-linear regression model was used to calculate their prediction strength.

**Results:**

The overall finish time correlated more with cycling and running times than with swimming times for both male and female IRONMAN^®^ age group triathletes (*r* = 0.88 and *r* = 0.89 for females; *r* = 0.89 and *r* = 0.90 for males, respectively). All correlation coefficients decreased with increasing age, which was more noticeable for the swimming discipline.

**Conclusions:**

Both cycling and running are more predictive than swimming in IRONMAN^®^ age group triathletes, where the correlation between the overall race times and the split times decreased with increasing age more in swimming than in cycling and running. These insights are useful for IRONMAN^®^ age group triathletes and their coaches in planning their IRONMAN^®^ race preparation and concentrating training on the more predictive disciplines.

## Key Points

Cycling and running split times correlated more with overall race times than swimming split times.

The correlation decreased with increased age, more pronounced in swimming than in cycling or running.

Female and male swimming performances were more similar than running and cycling performances.

## Background

Triathlon is a popular sport consisting of three disciplines – swimming, cycling and running – and different formats, such as sprint, Olympic distance, IRONMAN^®^ 70.3 (Half-IRONMAN^®^ distance), and IRONMAN^®^ 140.6 (Full- IRONMAN^®^ distance) [1], with the last one being the most demanding compared to the shorter distances regarding oxidative stress [2]. Since the first edition of IRONMAN^®^ Hawaii [3], the race has evolved into the IRONMAN^®^ World Championship, where both professional [4] and age group [5] triathletes have improved their performance over the years although the age of the fastest elite IRONMAN^®^ triathletes [4] and the fastest age group IRONMAN^®^ triathletes [6] increased in IRONMAN^®^ Hawaii.

In the last few years, several studies attempted to determine the most predictive split discipline for the IRONMAN triathlon overall race finish time using data from elite IRONMAN athletes [1, 7–9]. However, only one study investigated the IRONMAN^®^ age group triathletes [10]. For elite IRONMAN^®^ athletes, the cycling split seems to be the most predictive split discipline [7, 9]. Another study, however, concluded that cycling and running were the most decisive in an IRONMAN^®^ triathlon [8]. Furthermore, a study claimed that running is the most predictive split discipline in IRONMAN^®^ for elite triathletes [1]. For age group athletes, however, the fastest IRONMAN^®^ triathletes were the relatively fastest in running [10].

While the results seem contradictory for elite IRONMAN^®^ triathletes [1, 7–9], only one study investigating a sample of ~ 350,000 IRONMAN^®^ triathletes suggested that running was the most predictive split discipline for age group IRONMAN^®^ triathletes [10]. The disparate findings might be explained by the different performance levels of the athletes and the sample sizes of the subjects where professional IRONMAN^®^ athletes were compared to a large number of age group IRONMAN^®^ triathletes. In order to confirm the finding that running is the most predictive split discipline, a study with a considerably larger dataset is needed. Better understanding of the running contribution of split disciplines facilitates definition of training strategies to improve IRONMAN^®^ triathletes’ performance. That means that coaches and triathletes will be allowed to focus their efforts on improving a single discipline and thus achieve faster improvements for non-professional triathletes. However, performance in the running discipline will be influenced by previous efforts (e.g. how fresh I am when I start running) [11], thermoregulation [12], nutrition [13], and many other factors such as training and previous experience [14]. Therefore, running performance is not solely dependent on running training; it is influenced by the interactions of many other variables [15].

The aim of the present study was to investigate the importance of the three split disciplines swimming, cycling, and running on the overall race times in IRONMAN^®^ age group triathletes, in order to determine the most predictive discipline in IRONMAN^®^ for age group triathletes, and whether the importance of the split disciplines changes with increasing age. Based upon existing findings we hypothesized that running would be the most predictive split discipline in IRONMAN^®^ for age group triathletes of all age groups. We were also interested in whether the importance of the split disciplines would change with increasing age.

## Methods

### Ethics Approval

This study was approved by the Institutional Review Board of Kanton St. Gallen, Switzerland, with a waiver of the requirement for informed consent of the participants as the study involved the analysis of publicly available data (EKSG 01/06/2010). The study was conducted in accordance with recognized ethical standards according to the Declaration of Helsinki adopted in 1964 and revised in 2013.

### Dataset and Data Preparation

The race data (i.e., athlete´s sex, age, and country of origin, and the split times for swimming, running, cycling, transition times, and the overall race times) were downloaded from the official IRONMAN website (https://ironman.com) using a Python script. We considered only successful non-professional (i.e., age group athletes) finishers of all IRONMAN races recorded between 2002 and 2022 in the IRONMAN website. Data variables considered for statistical analysis were the time of each split discipline of the IRONMAN distance, including swimming (Swim Time), cycling (Bike Time), running (Run Time) and transition times (represented by transition 1 – (T1T) from swimming to cycling, and transition 2 – (T2T) from cycling to running) and overall race time (Finish Time), along with the triathlete´s age and sex. Age was recorded in 5-year age groups (i.e., 25–29, 30–34, 35–39, 40–44… 70–74 years) except for the first (18–24 years) and last (75+) groups. We combined all athletes older than 75 years into a single age group 75 + because the number of athletes in the higher age groups was very small.

### Statistical Analysis

Descriptive statistics are presented with absolute frequencies, mean, standard deviation, and maximum and minimum values, with all times displayed in hours or hh: mm: ss format. Data normality was verified by plotting the splits and full race times histograms for each sex. Boxplots of each split discipline time by age group and sex were built and displayed. Pearson and Spearman correlations between overall time and discipline times for each sex were calculated, first for the full sample and then for each age group. The correlation was interpreted according to Cohen criteria: <0.1 no correlation, 0.1–0.29 small, 0.3–0.49 medium, and > 0.5 large. An Ordinary Least Squares (OLS) Multiple Linear Regression model was built where the overall race time was a function of the split and transition times. The relationship between partial and full race times is also illustrated through scatter plots. All data processing and statistical analysis were done with Python (www.python.org/) in a Jupyter notebook (Google Colab) (https://colab.research.google.com/). The confidence interval was fixed at 95%.

## Results

**Fig. 1:**
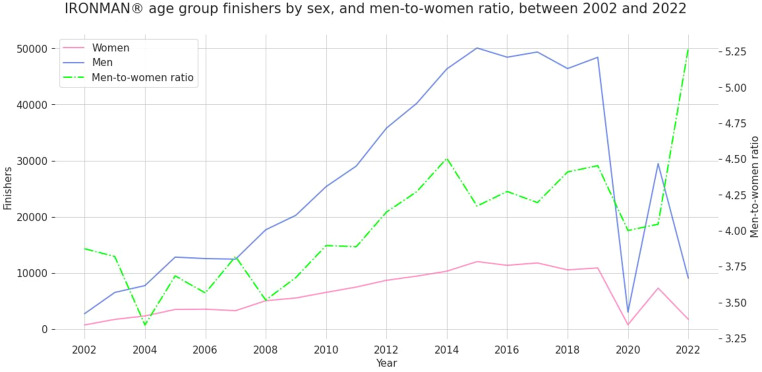
Finishes of female and male IRONMAN^®^ age group triathletes over the years between 2002 and 2022

Between 2002 and 2022, a total of 687,696 IRONMAN^®^ age group triathletes (553,608 men and 134,088 women) finished an official IRONMAN^®^ triathlon. Over the years, the number of female and male IRONMAN^®^ age group triathletes steadily increased. The exception was between 2019 and 2020, when the number of finishers abruptly decreased from annually ~ 60,000 finishers to ~ 6,000 finishers as a consequence of the COVID pandemic. The male-to-female ratio increased over the years, indicating a higher increase in male participation than female participation (Fig. 1).

**Fig. 2:**
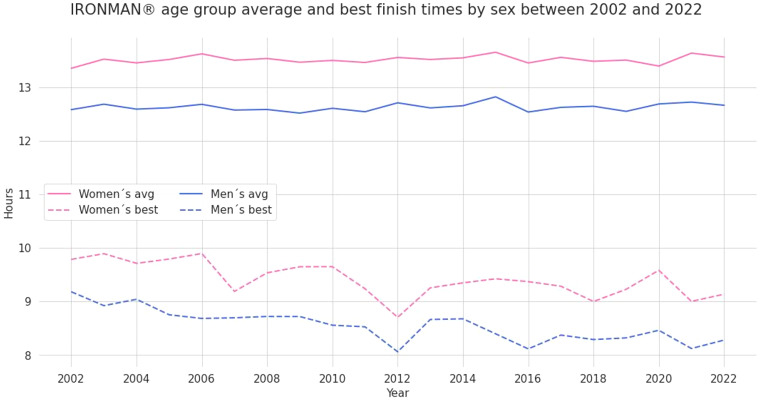
Trends of overall race times of female and male IRONMAN^®^ age group triathletes

Average race times of female and male IRONMAN^®^ age group triathletes remained stable over the years, whereas the best overall race times of female and male IRONMAN^®^ age group triathletes improved by approximately one hour between 2002 and 2022 (Fig. 2).

Most of the female and male IRONMAN^®^ age group triathletes were in the age group 40–44 years. On average, the fastest female IRONMAN^®^ age group triathletes were 25–29 years old, and the fastest male IRONMAN^®^ age group triathletes were 30–34 years old (Table 1).

**Table 1:** Overall race times for female and male IRONMAN^®^ age group triathletes

Overall race times (Women)					
Age Group	*n*	mean	sd	min	max
18–24	3409	13:16:04	01:40:08	09:35:50	16:59:42
25–29	13,568	13:07:35	01:41:06	09:00:49	17:14:54
30–34	22,689	13:11:43	01:41:47	09:07:09	17:38:06
35–39	24,647	13:19:36	01:41:19	09:00:00	17:34:20
40–44	26,505	13:30:14	01:39:32	09:18:57	17:50:43
45–49	20,848	13:43:29	01:36:48	09:00:08	17:23:49
50–54	13,757	14:01:25	01:34:16	09:43:47	17:57:34
55–59	5853	14:18:42	01:28:18	10:20:58	17:27:28
60–64	2106	14:46:36	01:21:46	08:42:17	17:16:58
65–69	577	15:17:54	01:07:27	12:02:51	16:59:13
70–74	116	15:43:20	00:55:42	13:24:04	16:59:31
75+	13	16:33:16	00:39:36	14:41:10	17:02:44
**Overall race times (Men)**					
**Age Group**	**n**	**mean**	**sd**	**min**	**max**
18–24	12,931	12:36:07	01:49:36	08:23:48	17:28:31
25–29	42,310	12:19:28	01:47:18	08:06:57	17:25:48
>30–34	78,919	12:15:21	01:46:16	08:07:22	17:44:01
35–39	101,705	12:20:21	01:45:19	08:12:43	17:27:31
40–44	116,384	12:31:19	01:43:48	08:21:39	17:50:33
45–49	94,384	12:44:21	01:43:01	08:19:20	17:32:48
50–54	61,987	13:03:32	01:42:17	08:03:41	17:28:07
55–59	28,367	13:27:46	01:40:35	08:31:11	17:39:05
60–64	11,413	13:53:40	01:37:04	09:10:50	17:33:02
65–69	3731	14:21:44	01:29:18	10:04:45	17:14:40
70–74	1214	14:59:52	01:14:59	08:32:40	17:02:02
75+	263	15:43:40	01:04:53	08:20:05	17:34:43

*Note* n (number of athletes); sd (standard deviation); min (minimum values); max (maximum values)

**Fig. 3:**
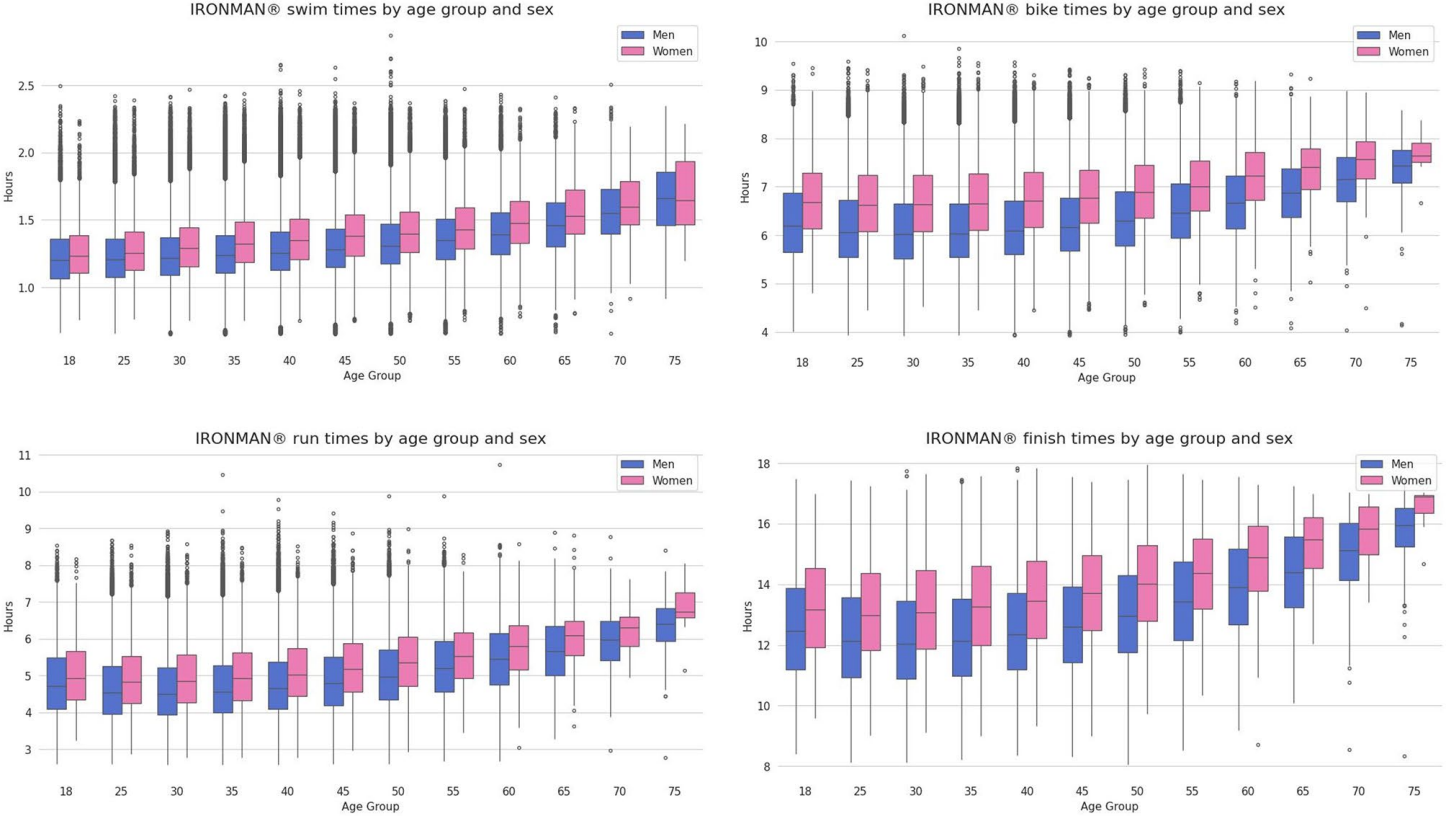
Split times in swimming, cycling, and running and overall race times for female and male IRONMAN^®^ age group triathletes in 5-year age groups

For all split disciplines (i.e., swimming, cycling, and running) and overall race times, female IRONMAN^®^ age group triathletes were always slower than male IRONMAN^®^ age group triathletes (Fig. 3).

**Fig. 4:**
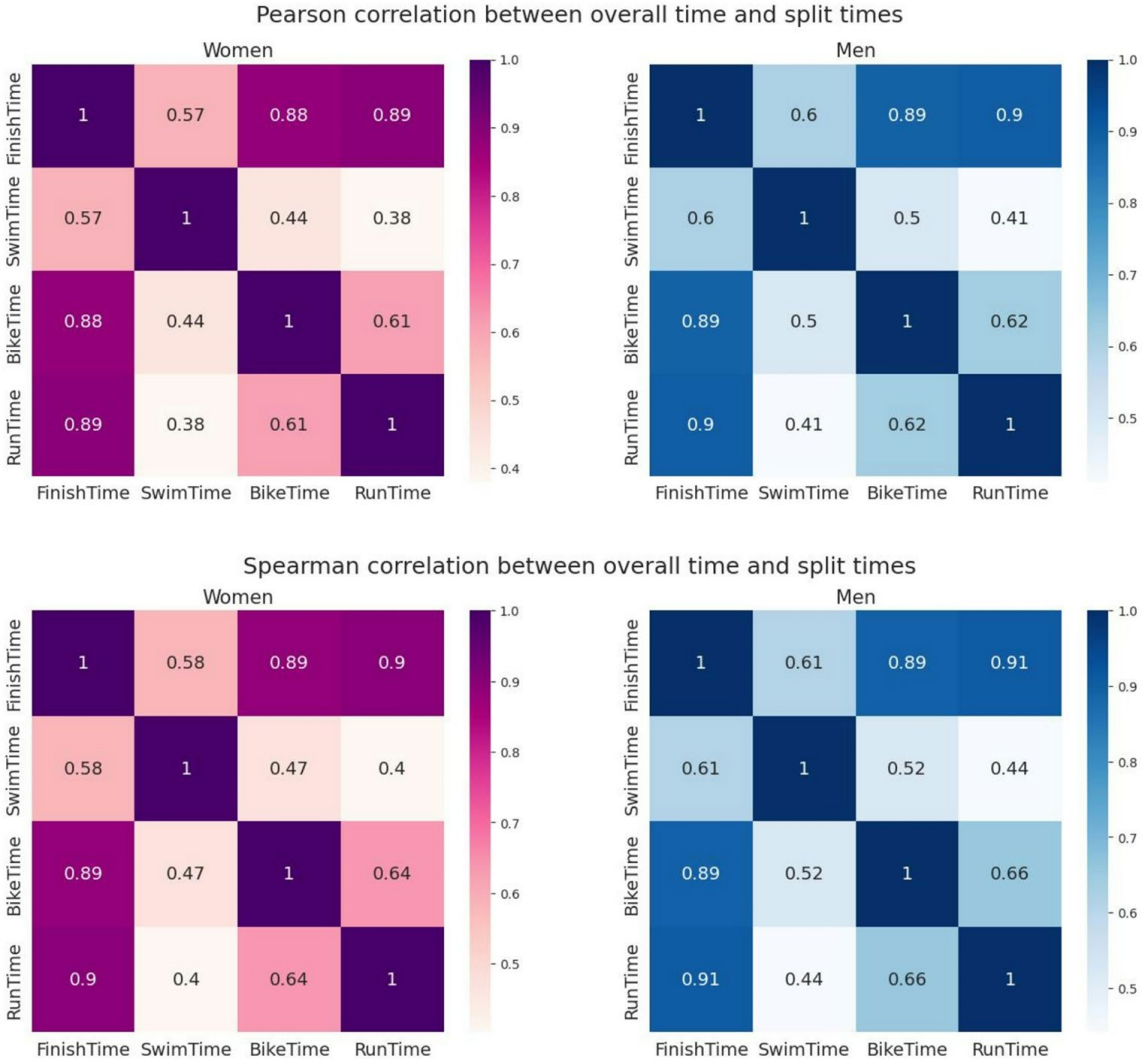
Pearson (upper panel) and Spearman (lower panel) correlations between split discipline and overall race times

The correlation matrixes show a larger relationship of the dependent variable ‘Finish Time’ with the variables ‘Bike Time’ and ‘Run Time’ than with the variable ‘Swim Time’ for both female and male IRONMAN^®^ age group triathletes (*r* = 0.88 and *r* = 0.89 for females; *r* = 0.89 and *r* = 0.9 for males, respectively) and for all age groups (Fig. 4). The results shown are almost identical, which indicates that the Spearman correlation does not detect any association beyond the linear relationship detected by the Pearson correlation.

**Fig. 5:**
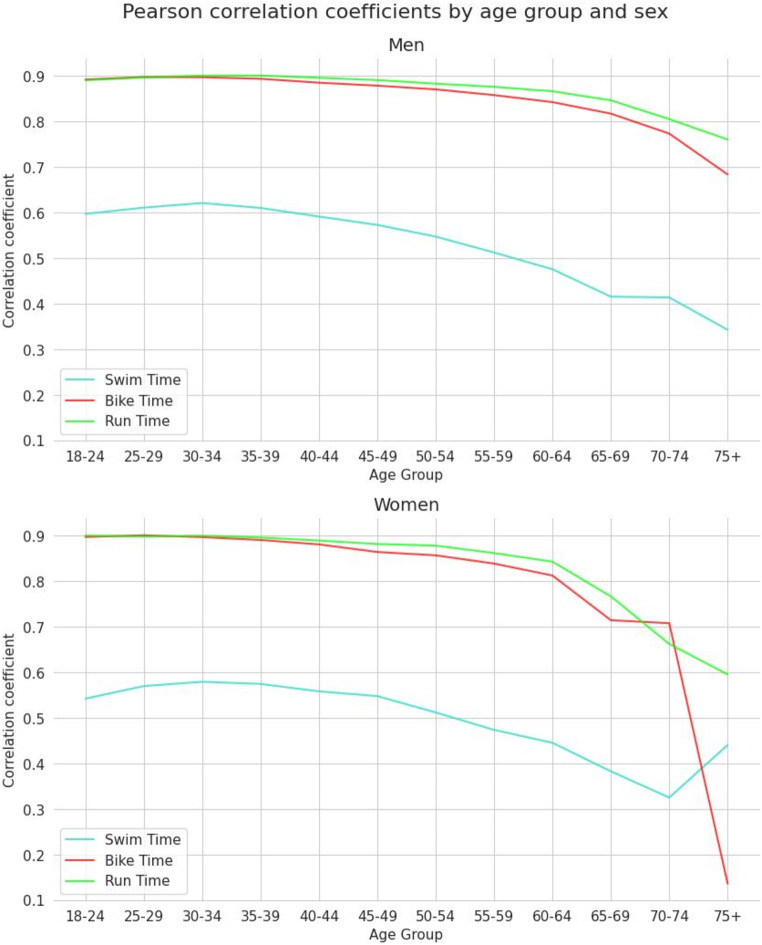
Pearson correlation coefficients for all age groups and both sexes (men in the upper panel, women in the lower panel)

When correlation was estimated based on the age group and sex, all correlation coefficients decreased with age. The correlation between the overall race time and the swimming time varied with age more notably than with the cycling or running times for both men and women (Fig. 5).

Table 2 shows the results of the MLR (Multiple Linear Regression) model of the race finish time as a function of the partial times and the transition times. As anticipated, the coefficients identified by the algorithm were 1 (or very near) for all variables, which indicates each second adds to the final race time. The perfect fit of the MLR regressor can also be concluded from the R-squared value of 1. The t-statistic value is a measure of how statistically significant each coefficient is, so we can see that swimming, cycling, and running times are one order of magnitude higher than transition times. Column P>|t| are the associated p-values (all zero). This is just an expected result, as cycling and running were much longer than swimming, so they contribute much more to the overall finish time. But still, all the variables are measured in the same units (seconds), so they all contribute equally to the race time.

**Table 2:** Multiple linear regression model results

Ordinary Least Squares Regression Results							
Dep. Variable:	FinishTime	R-squared:	1.000				
Model:	OLS	Adj. R-squared:	1.000				
Method:	Least Squares	F-statistic:	2.777e+09				
Date:	Sun, 15 Jan 2023	Prob (F-statistic): 0.00					
Time:	18:47:48	Log-Likelihood:	-3.6032e+06				
No. Observations:	687,696	AIC	7.206e+06				
Df Residuals:	687,690	BIC:	7.206e+06				
Df Model:	5						
Covariance Type:	nonrobust						
	**coef**	**std err**	**t**	**P>|t|**	**[0.025**	**0.975]**	
const	1.6910	0.481	3.515	0.000	0.748	2.634	
SwimTime	0.9999	7.7e-05	1.3e+04	0.000	1.000	1.000	
BikeTime	1.0000	2.7e-05	3.71e+04	0.000	1.000	1.000	
RunTime	0.9999	2.19e-05	4.57e+04	0.000	1.000	1.000	
Transition1Time	1.0004	0.000	3086.912	0.000	1.000	1.001	
Transition2Time	1.0005	0.000	3232.316	0.000	1.000	1.001	
Omnibus:	4879710.652	Durbin-Watson:		2.000			
Prob (Omnibus):	0.000	Jarque-Bera (JB):		13543447107810314.000			
Skew:	-829.097	Prob (JB):		0.000			
Kurtosis:	687499.856	Cond. No.		2.58e+05			

**Fig. 6:**
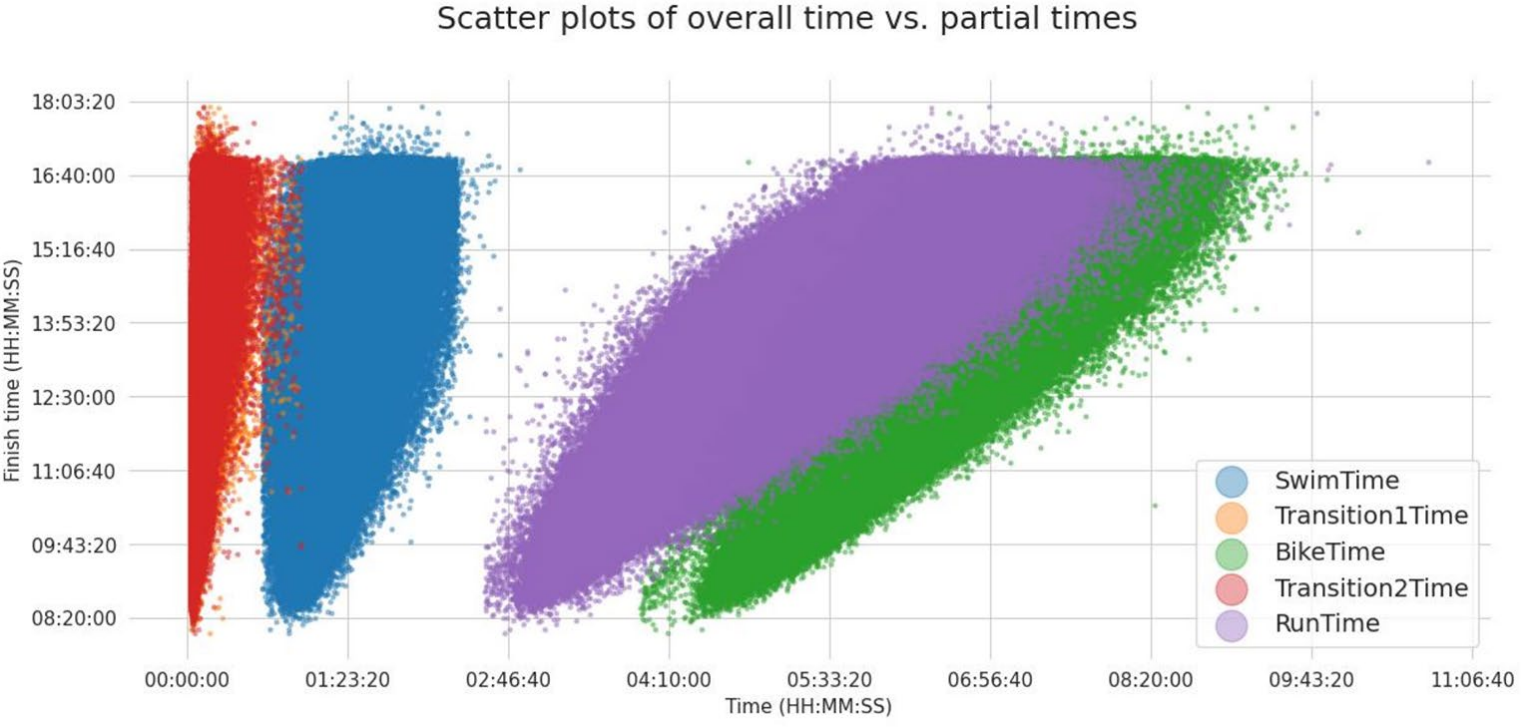
Scatter plots of overall race time with split discipline times

Figure 6 shows the scatter plots of the overall race time versus the split times with a sharp finish time limit at ~ 17 h. The charts also show the linear correlation with each variable and the collinearity of running and cycling times. We can also see the overall race time *versus* running time (green) and overall race time *versus* cycling time (orange) globes with a similar slope; however, the finish *versus* swimming scatter globe (blue) appears to have a larger slope which suggests that one second lost in swimming causes a larger loss in the overall race time than a second lost in running or in cycling. Since the x-axis extends further for the cycling and the running times, the swimming times appear compressed at the low end of the chart, appearing to contribute to the overall finish time more than they really do. The duration of the cycling and running splits is much longer than the swimming part so they naturally add more to the resulting time. But since the swimming split is much shorter, there are also “fewer swimming seconds” to lose. Every second counts and all add to the overall race time in the same proportion.

**Fig. 7:**
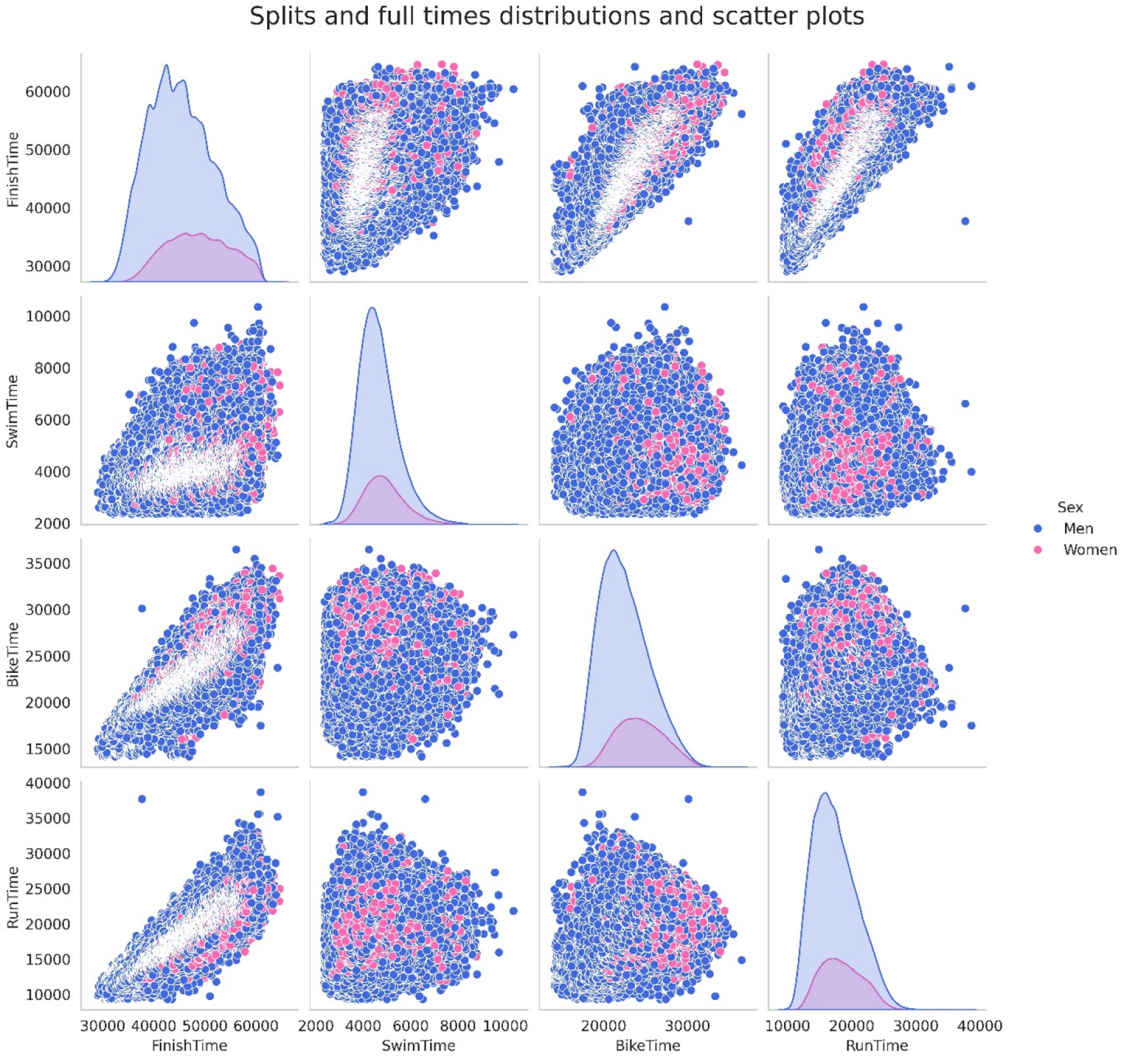
Pair plots of split discipline and overall race times (in seconds) by sex

Figure 7 shows the pair plots of split and overall race times by sex. The pair plots include scatter plots among different variables, along with each variable distribution in the diagonal. Worth noting is how much closer the men/women distributions of swimming times are when compared to the cycling or running times. As already observed earlier when analyzing correlations, this suggests that male and female swimming performances are more similar than running or cycling.

## Discussion

This study intended to determine the most predictive split discipline in IRONMAN^®^ age group triathletes with the hypothesis that running would be the most predictive split discipline for IRONMAN^®^ age group triathletes. The main findings were (i) for all age groups, the cycling and running times were more correlated with the overall race time than the swimming times, (ii) the correlation coefficients decreased with increasing age, and this effect was more pronounced in swimming than in cycling or running, (iii) female and male swimming performances were more similar than running and cycling performances and (iv) for all split disciplines and overall race time, male age group triathletes were always faster than female age group triathletes.

### Cycling and Running are More Predictive than Swimming

The first important finding was that both cycling and running were predictive for IRONMAN^®^ age group triathletes and we could not confirm our hypothesis that only running would be predictive. Our hypothesis was based on a study investigating a sample of more than 340,000 IRONMAN^®^ triathletes of all performance groups, including professional and age group triathletes competing between 2002 and 2015 in 253 different IRONMAN^®^ races [10]. In that study, IRONMAN^®^ triathletes were classified into nine different performance groups considering their overall race times (< 9 h, 9–10 h, 10–11 h, 11–12 h, 12–13 h, 13–14 h, 14–15 h, 15–16 h, and > 16 h) and 13 age groups (professionals, 18–24, 25–29, 30–34,… and 75–79 years). Regarding the importance of the split disciplines, IRONMAN^®^ triathletes in the fastest performance group were the relatively fastest in running and those IRONMAN^®^ triathletes in the slowest performance group were the relatively fastest in swimming and cycling. The most likely explanations that the former study found running to be the most predictive split discipline might be a different approach (i.e., evaluation of performance groups in the former study compared to age groups in the present) and the smaller sample size (half of the sample) of the former study (329,066 (80%) male and 81,815 (20%) female athletes) compared to the present study with 687,696 IRONMAN age group triathletes race records (553,608 from males and 134,088 from females).

Other studies considered only elite IRONMAN^®^ triathletes [1, 7–9], where only cycling [7, 9], cycling and running [8], and only running [1] were the most predictive split disciplines. Given that the current investigation included more than 680,000 IRONMAN^®^ age group triathletes, we must assume that both cycling and running are predictive in an IRONMAN^®^ triathlon. For practical applications, any athlete – independent of age – who will be cycling too fast will be slower in the run and any athlete saving energy during the cycling split might be able to achieve a fast marathon split [10].

The contribution of swimming to overall race time seems negligible. A study investigating the contribution of swimming, cycling, and running to overall race time in female and male Olympic distance and IRONMAN^®^ distance triathletes found that the average contribution of swimming (∼18%) was smaller than that of cycling and running for both distances and both sexes [6]. It may be suggested that swimming is of low importance regarding overall triathlon performance for shorter and longer race distances. However, it is known that at shorter distances with a vacuum allowance, swimming seems to be important for a good position in the peloton and thus saving energy for the race [16, 17].

Therefore, coaches might advise future IRONMAN^®^ triathletes to conserve energy when swimming, even swimming breaststroke. This might help save energy for the following cycling and running splits. They also may reduce time spent on training for swimming in comparison to cycling and running. Moreover, it has been suggested for short-distance events that swimming at a reduced intensity may result in enhanced performance in the subsequent disciplines [16]. Also, other studies have reported that positioning in the leading packs during the swimming has been shown to have a strong relationship with achieving a better result in Olympic distance triathlon [18–20] However, it is important to highlight that the technique of specialized and elite swimmers is more efficient compared to the swimming technique of triathletes [21, 22]. Also, high training volumes are required for swimming and triathletes cannot dedicate as much time training for each discipline as a specialized swimmer, cyclist, or runner. It has been shown for recreational (age group) IRONMAN^®^ triathletes that high volumes of training of more than 20 h per week, when performed 40 days before a race, impaired performance compared to lower volumes of training of up to 14 h per week [14].

### Changes of Correlation Coefficients with Increasing Age

A second important finding was that the contribution of split disciplines decreased with increasing age since the correlation coefficients decreased with increasing age. We found that the decrease of the correlation coefficients started at the age of ~ 30–35 years (Fig. 5), which is coincidental with the age of peak IRONMAN^®^ triathlon performance [23]. After this age, the decline of the correlation coefficients was relatively linear, similar to the age-related performance decline in IRONMAN^®^ triathlon [24]. This finding refers to male triathletes. Regarding female triathletes, the age of peak IRONMAN^®^ triathlon performance is very similar where it has been reported that women and men peak at a similar age of 32–33 years in an IRONMAN^®^ triathlon with no sex difference [25]. However, the age-related performance decline differs between female and male IRONMAN^®^ triathletes. It has been shown that the age-related performance decline started in female IRONMAN^®^ triathletes in age group 25–29 years in swimming and in age group 30–34 years in cycling, running, and overall race time, whereas it started in male IRONMAN^®^ triathletes in age group 25–29 years in swimming and in age group 35–39 years in cycling, running, and overall race time [24].

An interesting aspect was that, with increasing age, the contribution of swimming decreased in a more pronounced way compared to cycling and running. The aspect of the contribution of swimming, cycling, and running split times to overall race time has been investigated for different race distances [8, 26]. Figueiredo et al. investigated the changes in the contribution of swimming, cycling, and running to overall race time in female and male Olympic distance and IRONMAN^®^ distance triathletes over 26 years of races. For the Olympic distance triathlon, split times in swimming and running decreased, whereas split times in cycling remained stable over the years. In the IRONMAN^®^ distance triathlon, split times in swimming remained stable over the years, but the times in cycling and running decreased. During the 26-year period, the contribution of swimming (women and men) decreased and the contribution of cycling (men) increased in the Olympic distance triathlon. For the IRONMAN^®^ distance triathlon, the contribution of swimming and cycling undulated, whereas the contribution of running decreased in males [8]. Stevenson et al. compared the sex differences for the Sprint distance, the Olympic distance, and the IRONMAN^®^ 70.3 distance triathlon for age group triathletes. The sex differences in performance were the smallest in cycling for the Sprint distance and the IRONMAN^®^ 70.3 distance, whereas for running, the sex difference was the smallest in the Olympic distance triathlon [26]. However, to date, this is the first study investigating the aspect of age regarding the influence on split performance in the IRONMAN^®^ triathlon. Future studies need, however, to investigate in more detail the contributions of split disciplines with increasing age in triathlon races of different distances such as the Sprint distance, the Olympic distance, or the IRONMAN^®^ 70.3 distance.

### Differences between Split Performances by Age Groups

A third important finding was that male and female swimming performances were more similar than running and cycling performances. In other words, female IRONMAN^®^ age group triathletes achieved a similar swimming performance as male IRONMAN^®^ age group triathletes. It has been observed that female age group freestyle swimmers in pool [27] and open-water [28] swimming can perform similarly to male age group swimmers. In freestyle pool swimming, females were not slower than males in age groups 80–84 to 85–89 years [27] and in 3000 m open-water swimming, race times were similar for both females and males in age groups 75–79 and 85–89 years [28].

Regarding running, elderly females could also close the gap with elderly males in different running distances [29, 30]. In running races covering 5 km, 8 km, 10 km, 10 miles, 20 km, half-marathon, 25 km, 30 km, marathon, 50 km, 50 miles, 100 km, 100 miles, 12 h, 24 h, 48 h and 144 h, females could reduce the gap to males with increasing age, but not with increasing the length/duration of the running event [29]. A comparison between 50 miles and 100 miles of ultra-marathon running showed that the sex differences decreased with increasing age and were smaller in 100-mile than in 50-mile races [30].

Sex differences have also been investigated in cycling [31, 32]. Generally, males are faster than females in long-distance cycling [33]. However, as age and/or distance/duration increase, females can come closer to achieving parity with males [34]. An analysis of time-limited ultra-cycling races covering 6 h, 12 h and 24 h showed that the sex differences in cycling speed decreased with increasing duration of the races and with increasing age [31]. In distance-limited ultra-cycling races (100 miles, 200 miles, 400 miles, and 500 miles), the sex difference decreased with increasing age, and men were faster than women in 100- and 200-mile races, but no sex differences were identified for the 400- and 500-mile races [32].

The reasons behind females gaining ground on males as they age and cover longer distances remain unclear. The participation of females in ultra-endurance races is an important aspect since the percentage of females is generally low in these races [35]. Lower female participation relative to male participation overestimates the age-related performance decline, especially in very old females [36]. A small sex difference in ultra-marathon running performance is most likely due to a low number of participants in a specific age group than due to outstanding physiology [37]. An analysis of 20 different ultra-marathons covering 45 to 160 km showed that the sex difference in running was lower in the longer distances and largest when there were fewer females and males in a specific race [38]. A study analyzing more than 1 million race records of female and male runners competing in race distances (i.e., 5 km, 10 km, half-marathon, marathon, and ultra-marathon) showed that the male-to-female ratio declined with increasing race distance and elderly female ultra-marathoners (75 years and older) displayed a performance difference of less than 4%. It was assumed that this low sex difference was due to the presence of highly selected outstanding elderly female performers [39]. Most likely, the finding that women are narrowing the performance gap with men as they age is due to participation, rather than performance.

### Male Age Group Triathletes Were Always Faster than Female Age Group Triathletes

A last important finding was that male IRONMAN^®^ age group athletes were faster in all split disciplines and in overall performance. It is well-known that male IRONMAN^®^ triathletes are faster than female IRONMAN^®^ triathletes. On average, for non-elite (age group) IRONMAN^®^ triathletes, the sex difference is ~ 12% in swimming, ~ 15% in cycling and ~ 18% in running [40]. Also, in IRONMAN^®^ 70.3 races covering half of the IRONMAN^®^ distance, men were faster than women in all split disciplines and all age groups [41]. Overall, male triathletes are faster than female triathletes for all distances shorter than the IRONMAN^®^ triathlon from the Sprint distance, the Olympic distance, and the IRONMAN^®^ 70.3 distance [26].

### Limitations

Although the dataset is very large, this kind of analysis has some limitations. Specific aspects such as the pre-race preparation [42], training [14], nutrition [43, 44], and dropouts due to overuse injuries [45, 46] were not considered in this data analysis. In addition, despite the large use of secondary datasets to improve and translate theoretical and practical knowledge, the accuracy of the data (i.e., the distance in each event) should be considered a limitation. Environmental variables, including the place of competition, the characteristics of the course and the natural environment (i.e., humidity, wind, temperature), should be considered in future studies to identify how these different factors influence the split times of athletes competing in different settings.

## Conclusion

In summary, in IRONMAN^®^ age group triathletes, both cycling and running are more predictive of overall race finish time than swimming. The correlation decreases more with increasing age in swimming than in cycling and running. Furthermore, female and male swimming performances were more similar than running and cycling performances. Based on our results IRONMAN^®^ age group triathletes need to focus more on cycling and running than swimming training. These insights are useful for IRONMAN^®^ age group triathletes and their coaches in planning their IRONMAN^®^ race preparation.

## Data Availability

For this study, we have included official results and split times from the official IRONMAN^®^ website (https://ironman.com). The datasets used and/or analysed during the current study are available from the corresponding author on reasonable request.

## Abbreviations

OLS: Ordinary Least Squares
MLR: Multiple Linear Regression
